# Hemosuccus Pancreaticus: A Great Masquerader in Patients with Upper Gastrointestinal Bleeding

**DOI:** 10.7759/cureus.3785

**Published:** 2018-12-27

**Authors:** Faisal Inayat, Nouman Safdar Ali, Maryam Khan, Ahmed Munir, Waqas Ullah

**Affiliations:** 1 Internal Medicine, Allama Iqbal Medical College, Lahore, PAK; 2 Internal Medicine, Jinnah Sindh Medical University, Karachi, PAK; 3 Internal Medicine, Services Institute of Medical Sciences, Lahore, PAK; 4 Internal Medicine, Abington Jefferson Health, Abington, USA

**Keywords:** hemosuccus pancreaticus, gastrointestinal bleeding, diagnostic challenge, side-viewing duodenoscope

## Abstract

Hemosuccus pancreaticus is a rare but life-threatening cause of upper gastrointestinal bleeding through the main pancreatic duct. This clinical entity is a difficult diagnosis due to its rarity, intermittent nature of the hemorrhage, and peculiar clinical presentation. It is still considered a surgical problem but advances in medical therapy may enable clinically stable patients to undergo less-invasive angiographic embolization. We chronicle here a unique case of hemosuccus pancreaticus in a patient presenting with melena who could not be diagnosed on multiple standard forward-viewing esophagogastroduodenoscopies and computed tomography angiography. Eventually, side-viewing duodenoscope identified the intermittent bleeding through the ampulla of Vater. This paper illustrates that clinicians should be vigilant for this etiology, especially in patients with intermittent crescendo-decrescendo abdominal pain, acute gastrointestinal hemorrhage, and elevated serum lipase levels. A multidisciplinary team approach with the centralization of gastrointestinal bleed services and a well-established management protocol is of paramount importance to reduce the morbidity and mortality of this disorder. Additionally, this article serves to outline our current understanding of the epidemiology of and risk factors for hemosuccus pancreaticus, the pathophysiology of this disease, and currently available approaches to diagnosis and treatment.

## Introduction

Hemosuccus pancreaticus is an unusual etiology that is characterized by intermittent upper gastrointestinal bleeding from the ampulla of Vater into the duodenum [1]. It predominantly occurs in the settings of chronic pancreatitis, aneurysms of peripancreatic vessels, and pancreatic tumors [2,3]. Although endoscopy and radiologic investigations are commonly used, computed tomography angiography (CTA) is the gold standard diagnostic modality [4]. Interventional radiology procedures like endovascular embolization are used to manage hemodynamically stable patients [5]. Surgical intervention is a feasible treatment option in cases where interventional radiologic techniques fail or among hemodynamically unstable patients [6]. As atypical presentations of gastrointestinal bleeding commonly pose a diagnostic challenge, it is imperative to consider hemosuccus pancreaticus in the differentials, especially in patients without an obvious cause of bleeding.

We describe here a case of the patient who was admitted to our hospital with melena. He underwent multiple upper endoscopies and cross-sectional imaging, but bleeding focus could not be identified. Subsequently, side-viewing duodenoscope revealed intermittent bleeding via the ampulla of Vater. However, the patient refused the eventual treatment, which resulted in the fatal outcome. Furthermore, this article reviews the pertinent medical literature for clinical features, pathogenesis, and management of hemosuccus pancreaticus with a special emphasis on the need for prompt diagnosis. This case has previously been presented as an abstract (Abstract: Inayat F, Iqbal S, Khan ZH, Hussain Q, Lodhi HT, Zafar F, Munir A, Hurairah A, and Quainoo C. Hemosuccus Pancreaticus: Side-viewing Endoscopic Diagnosis of a Rare but Fatal Cause of Gastrointestinal Hemorrhage. Annual Scientific Meeting, American College of Gastroenterology; October 05-10, 2018, Philadelphia, Pennsylvania).

## Case presentation

A 70-year-old male presented to our medical center with one episode of “black tarry stools” in the morning. Three weeks prior to this admission, the patient underwent esophagogastroduodenoscopy (EGD), which revealed a 6-mm, clean-based ulcer at the gastroesophageal junction without active bleeding. Colonoscopy, performed during the same visit, was significant for mild diverticulosis and small nonbleeding hemorrhoids. He had a medical history of chronic active alcohol abuse, prior gastrointestinal bleeding, hypertension, diabetes mellitus type 2, and non-small-cell lung cancer (T2N0M0; status post-lobectomy, 18 years ago). The patient had been consuming 3-4 drinks of liquor daily; however, he was non-smoker and drug-free. He was under therapy with oral iron sulfate, metformin, pantoprazole, enalapril, and multivitamins. Review of the systems was significant for fatigue, malaise, and confusion. His vitals included blood pressure 137/81 mm Hg, heart rate 108 beats per minute, temperature 36.9°C, respiratory rate 16 per minute and oxygen saturation 100% on room air. On physical examination, he was in no acute distress but appeared lethargic. The cardiopulmonary examination was inconclusive for abnormalities. The abdomen was soft and non-tender without organomegaly.

He underwent extensive diagnostic workup. The details of his laboratory studies are provided in Table 1.

**Table 1:** Laboratory results of the patient with respective reference ranges.

Laboratory parameter	Patient result	Reference range
White cell count	8.6	4.5-11.0/uL
Hemoglobin	8.7	13-18 g/dL
Mean corpuscular volume	82.1	80-96 fL/red cell
Platelets	368 × 10^3^	150–450 × 10^3^
International normalized ratio	1.2	<1.1
Alanine transaminase	26	7-56 U/L
Aspartate aminotransferase	22	10-40 U/L
Alkaline phosphatase	278	44-147 U/L
Total bilirubin	0.5	0.1-1.2 mg/dL
Serum lipase	209	0-50 U/L
Serum amylase	101	23-85 U/L
Blood urea nitrogen	35	7-20 mg/dL
Creatinine	1.0	0.4-1.2 mg/dL

**Figure 1:**
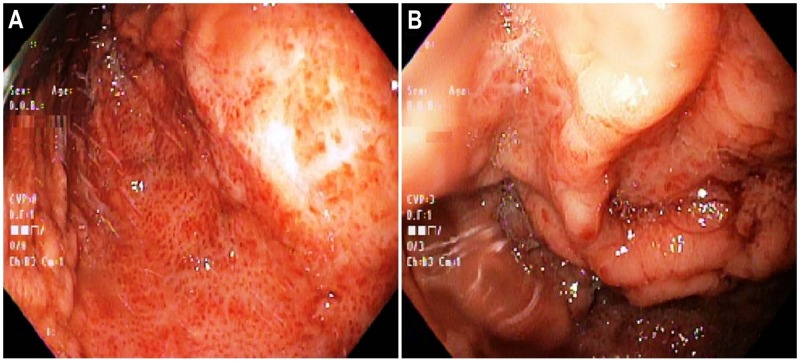
Upper endoscopy performed at presentation. (A) Nodular, edematous and erythematous mucosa with petechial hemorrhages most prominent in the gastric fundus and body. (B) Several questionable prominences of underlying vasculature were evident.

Chest radiograph showed left-sided thoracotomy with chronically elevated left hemidiaphragm but no airspace opacity, effusion, or pneumothorax. He was started on intravenous proton-pump inhibitor therapy for upper gastrointestinal bleeding. Subsequently, EGD showed nodular, edematous and erythematous mucosa with petechial hemorrhages in the gastric fundus and body, with questionable prominence of underlying vasculature (Figure 1).

**Figure 2:**
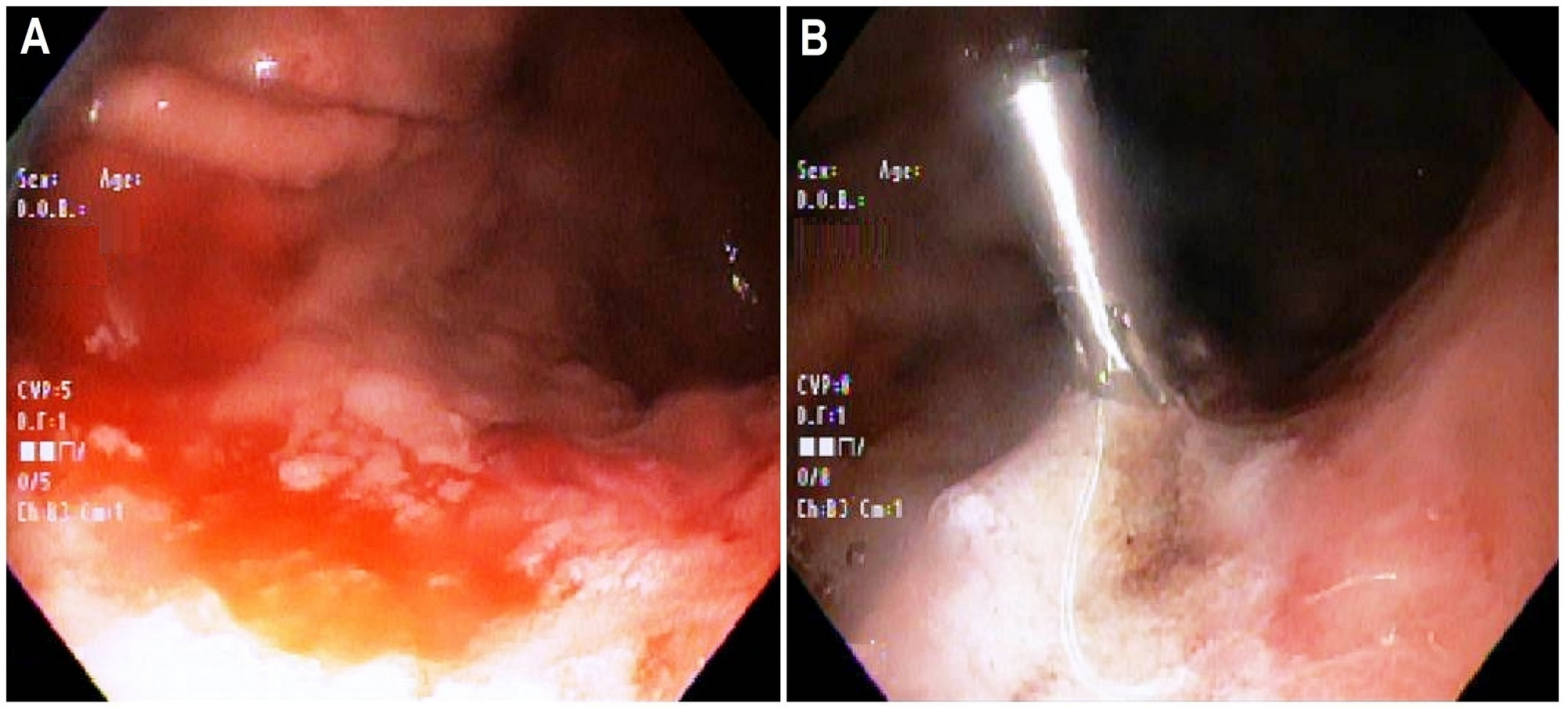
Repeat upper endoscopy performed one week after admission. (A) Clots and fresh blood were noted in the proximal body of the stomach. (B) An actively blood-oozing pinpoint area, likely a Dieulafoy’s lesion, was identified under a removed clot in the gastric body; hemostasis was secured using endoscopic combination therapy consisting of epinephrine, electrocoagulation, and hemoclip application.

The endoscopy was inconclusive for an active bleeding focus; duodenum appeared normal. He also received one unit of packed red blood cells. During the course of hospitalization, he continued to experience black-colored stools. His hemoglobin demonstrated a gradual downward trend, prompting the need for a second endoscopic evaluation. On day 7 of admission, repeat EGD revealed frank fresh blood along with blood clots in the proximal body of the stomach. The clots were irrigated, suctioned, and removed with Roth net. A pinpoint area under one of the removed clots was actively oozing, which mimicked a Dieulafoy’s lesion; primary hemostasis was achieved with a combination therapy using epinephrine injection, cautery, and hemoclip application (Figure 2).

**Figure 3:**
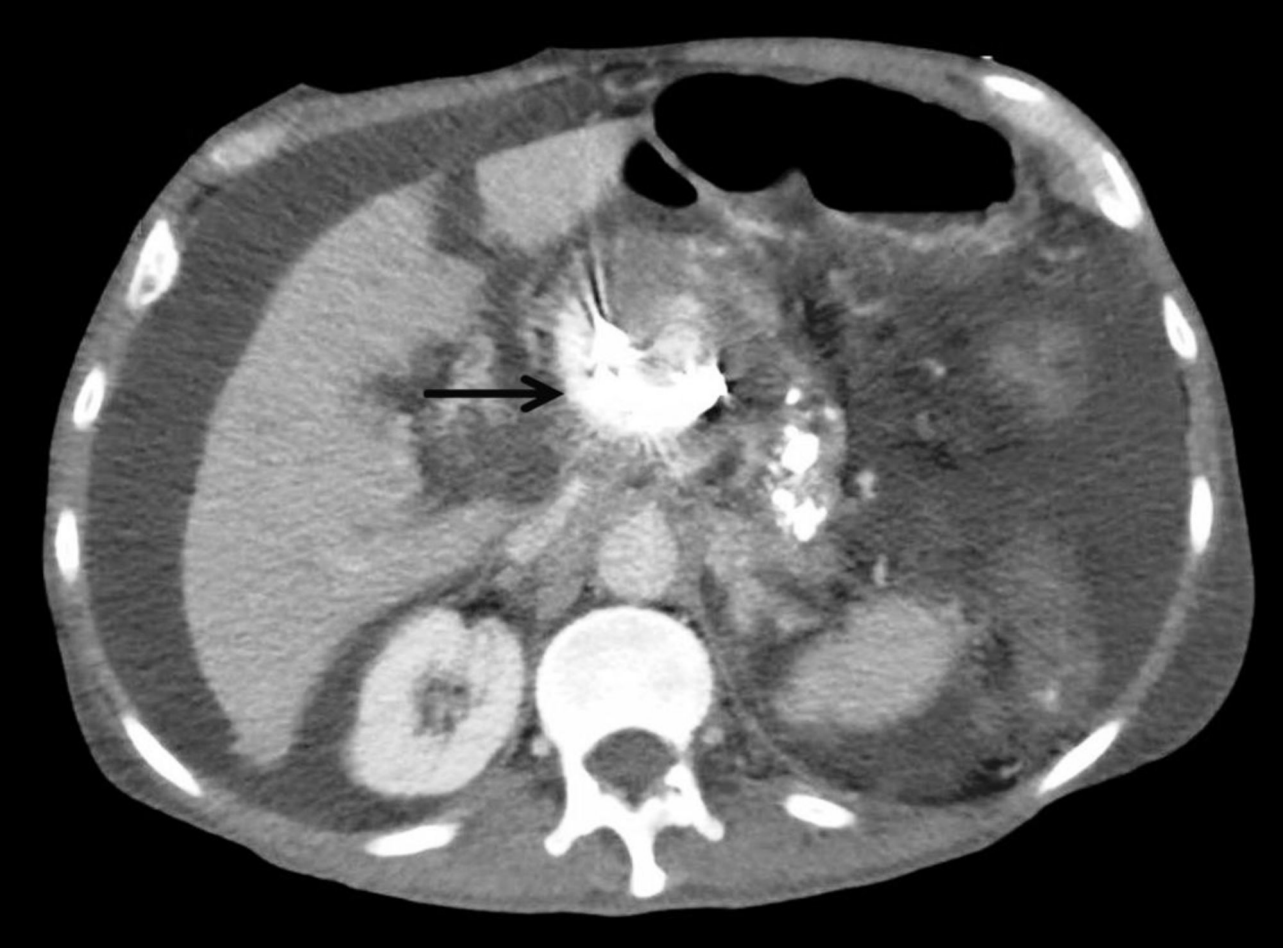
Computed tomography scan of the abdomen with pancreatic imaging protocol showing coil embolization (arrow) of the pseudoaneurysm in the head of the pancreas.

On day 10 of admission, laboratory studies showed an acute elevation of liver enzymes (alanine transaminase 1096 IU/L; aspartate aminotransferase 123 IU/L; alkaline phosphatase 47 IU/L; and total bilirubin 1.3 mg/dL). Drug-induced liver injury and obstructing lesion were considered plausible. Computed tomography (CT) with pancreatic imaging protocol revealed a large 4.2 x 2.9-cm pseudoaneurysm, likely arising from the gastroduodenal artery, located in the head of the pancreas, with evidence of moderate intrahepatic ductal dilatation and portal vein obstruction. Given the large size with compressive features of the surrounding structures, embolization of the pseudoaneurysm utilizing 11 coils with thrombin/Gelfoam injection was performed (Figure 3).

A two-day post-procedure abdominal ultrasound showed an increase in the size of the pseudoaneurysm, measuring 5.6 x 5.8 cm. Interventional radiology then repeated the embolotherapy with thrombin injection. In the next few days, he continued to report both melena and bright red blood per rectum, but his hemoglobin remained stable. On day 19 of admission, third EGD was performed, which was unremarkable for an active bleeding spot. His hemoglobin started to drop but another endoscopic evaluation was not recommended as last EGD was unremarkable. Therein, a bleeding scan showed an equivocal left-flank bleeding focus, not suggestive of a lower gastrointestinal collection, but possibly a small bowel focus with low-grade extravasation. Subsequently, his hemoglobin dropped to 6.3 g/dL, with no signs of overt gastrointestinal hemorrhage. On day 27 of admission, abdominal paracentesis ruled out hemoperitoneum and CTA abdomen showed possible hemorrhage in the left flank outside the gastrointestinal tract, possibly due to an infarcted spleen.

**Figure 4:**
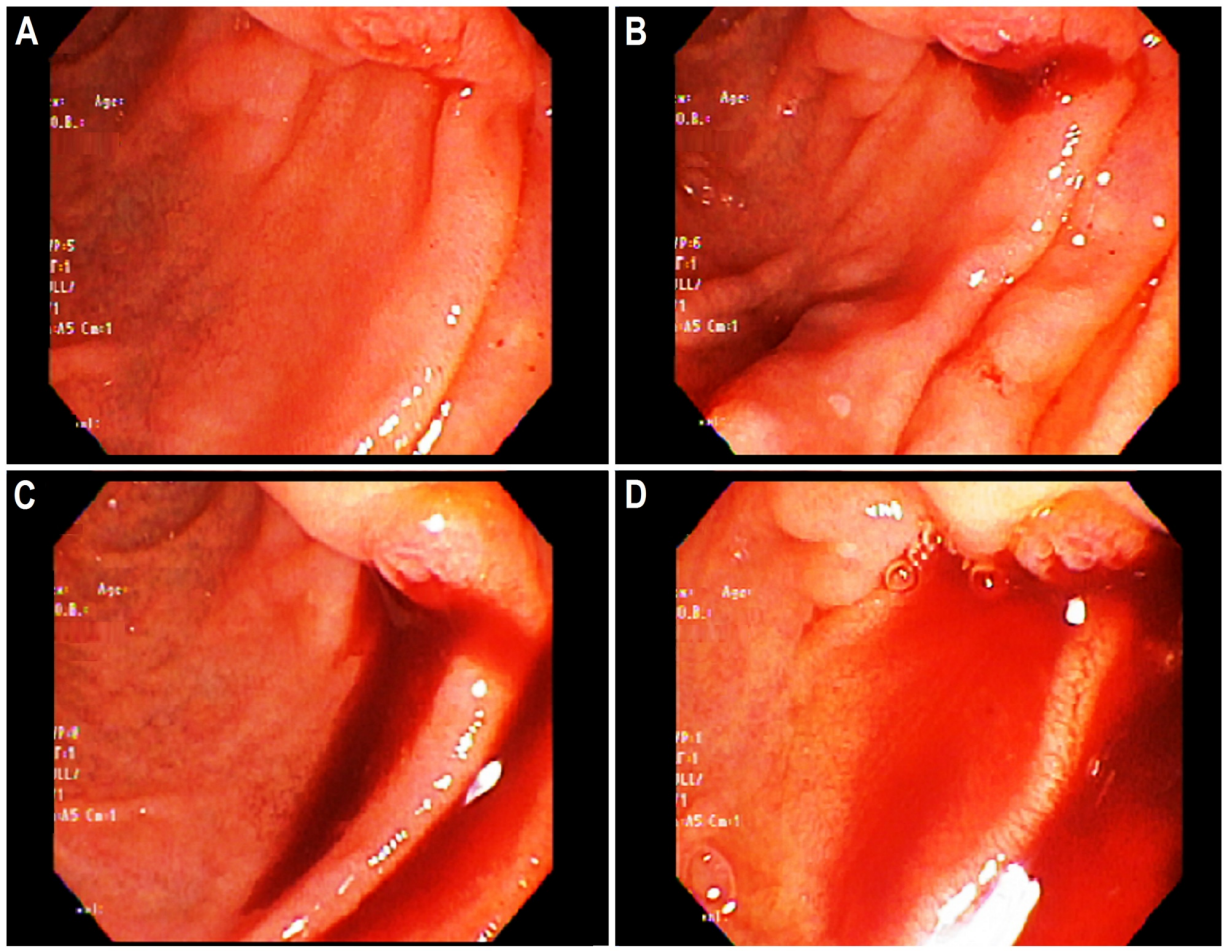
The sequential findings of side-viewing upper endoscopy performed four weeks after admission revealing hemosuccus pancreaticus. (A) Extremely negligible oozing of blood from the ampulla of Vater on initial view. (B) Improved visibility of bleeding due to blood extravasation from the ampulla of Vater on the subsequent view. (C) Rapid oozing of the blood from the same spot within the next few seconds, confirming hemosuccus pancreaticus. (D) Frank blood accumulated in the duodenum in next one minute.

After a discussion with surgery and gastroenterology teams, a repeat EGD with a side-viewing duodenoscope was planned. It initially demonstrated an extremely small blood-oozing spot from the ampulla of Vater. The blood extravasation increased shortly after first pinpointing the source. Over the next few seconds, the blood started to ooze at a rapid rate. In the next one minute, frank intermittent bleeding through the ampulla of Vater into the duodenum was identified (Figure 4).

These findings were consistent with hemosuccus pancreaticus, likely due to the bleeding from the pseudoaneurysm of the gastroduodenal artery. After the precise etiology establishment, surgical intervention was considered due to prior failed attempts of embolization. However, there was a high risk of perioperative mortality given the patient’s comorbid conditions, poor clinical and performance status and complexity of the anatomical location of pseudoaneurysm. Therefore, a conservative approach was preferred by the patient and his family. On the other hand, he continued to experience intermittent hemorrhage requiring blood transfusions with episodes of hemodynamic instability. An urgent embolization of the pseudoaneurysm was planned. However, the patient and his family refused to undergo therapeutic procedures. The patient continued to worsen clinically over the next few days but he wished not to be resuscitated. He died in a couple of days following hemodynamic instability.

## Discussion

In 1931, Lower and Farrell first identified upper gastrointestinal bleeding through the main pancreatic duct in a patient with ruptured splenic artery aneurysm [1]. However, Sandblom first coined the term *hemosuccus pancreaticus* in 1970 and reported three patients with bleeding pseudoaneurysms [2]. This condition remains extremely rare with approximately 150 cases reported thus far [3]. Based on the limited data, it has been estimated that 1 in 1500 cases of upper gastrointestinal hemorrhage is due to hemosuccus pancreaticus. It is commonly observed in males (gender ratio: 7:1) in their fifth or sixth decades of life, with a history of long-term alcohol consumption [3].

The clinical presentation in these patients is varied but a triad of epigastric pain, gastrointestinal bleeding and hyperamylasaemia has been encountered in a vast majority of patients [4]. Melena is the most frequently observed form of upper gastrointestinal bleeding followed by hematemesis. The bleeding is intermittent and usually not very severe even though it has an arterial origin. The epigastric pain predominantly results from increased pressure in the pancreatic duct due to blood clots-related obstruction. In these patients, serum amylase elevates secondary to the concurrent presence of acute pancreatitis [4]. Nausea, vomiting, unintentional weight loss, a palpable aneurysmal mass, hemodynamic compromise, and/or jaundice due to bile duct obstruction by pancreatic head masses are among other notable clinical features [5]. In regards to liver function testing, increased serum bilirubin secondary to pancreaticobiliary reflux may be noted. This patient did not report any symptoms of acute or chronic pancreatitis even though his initial laboratory studies showed elevated serum amylase and lipase levels.

Hemosuccus pancreaticus has been reported in association with conditions like pancreatitis, pancreatolithiasis, pancreatic neoplasms, and splenic rupture [6]. Similarly, ruptured pseudoaneurysms of the surrounding vessels, including splenic, gastroduodenal, pancreaticoduodenal, superior mesenteric and hepatic arteries have commonly been reported as sources of bleeding [7]. In cases of acute pancreatitis, arterial wall rupture due to necrosis and/or enzymatic destruction causes the hemorrhage [8]. Pseudocysts, arterial aneurysms, and pseudoaneurysms develop over time in patients with chronic pancreatitis. Pseudocysts erode the arterial wall as a result of chronic inflammatory injury and become pseudoaneurysms. In approximately 10% of cases of chronic pancreatitis, pseudoaneurysms and aneurysms rupture and bleed into the pancreatic duct through fistulous tracts [9,10]. The common and uncommon associations of hemosuccus pancreaticus have been summarized in Table 2.

**Table 2:** Literature review on associations of hemosuccus pancreaticus. RCC: Renal cell carcinoma; PD: Pancreatic duct; AVM: Arteriovenous malformation; ERCP: Endoscopic retrograde cholangiopancreatography; EUS: Endoscopic ultrasound; FNA: Fine-needle aspiration.

Cause	Type	Pathogenesis	Incidence
Pancreatitis	Acute, chronic	Necrosis of arterial wall, rupture of pseudoaneurysm, pancreatic stones	Most common
Tumor	Pancreatic carcinoma, serous cystic neoplasm, neuroendocrine tumor, microcytic adenoma, metastatic RCC	Tumor hemorrhage through PD	Common
Vascular	Aneurysm, pseudoaneurysm, AVM	Rupture into PD	Common
Iatrogenic	ERCP, EUS-guided FNA, pancreatic stenting	Penetration of peripancreatic arteries	Rare
Congenital	Pancreas divisum, heterotopic pancreas	Unclear	Rare
Infection	Pancreatic brucellosis, syphilis	Erosion of mycotic or syphilitic aneurysm into PD	Rare
Trauma	Blunt, penetrating	Rupture of post-traumatic pseudoaneurysm	Rare

This disease frequently poses a diagnostic challenge. Although endoscopy is the initial investigation of choice for upper gastrointestinal bleeding, only about 30% of cases of hemosuccus pancreaticus can be diagnosed by this procedure. Endoscopic evaluation is of prime importance to exclude more common causes of upper gastrointestinal hemorrhage, including acute erosive gastritis, peptic ulcer disease, and esophageal and gastric fundus varices as well as relatively uncommon causes like Dieulafoy’s lesion [11-13]. Advanced endoscopic techniques such as endoscopic retrograde cholangiopancreatography (ERCP) and endoscopic ultrasound (EUS) are utilized for better visualization of the source of bleeding [14]. In order to detect bleeding from the pancreatic duct into the duodenum, forward-viewing endoscopic techniques may have relatively lower diagnostic yield. Duodenoscopes used for ERCP are side-viewing endoscopes that efficiently locate the hemorrhage in the pancreatic duct and facilitate the detection of bile duct pathologies in difficult-to-diagnose cases [15].

CT abdomen with contrast is a good investigation in these patients as it not only identifies the pancreatic parenchymal etiology but also demonstrates radiological signs of peripancreatic cystic and aneurysmal lesions [16]. However, the pathognomonic clotted blood in the pancreatic duct, described as the sentinel clot, is usually not identified. CT may also indicate the concomitant aneurysmal artery opacification or contrast persistence within a pseudocyst [16]. It is particularly notable that these radiologic features can only suggest the diagnosis. Thus, the gold standard diagnostic modality remains selective CTA with a sensitivity of 96% [17]. The vascular abnormality can be located and further management can also be gauged accordingly by using CTA [17]. However, this patient was unique in this regard as diagnostic investigations like initial endoscopy, early forward-viewing ultrasound and CTA could not identify any vascular abnormality in the duodenum, culminating in an inadvertent delay in the diagnosis. Side-viewing duodenoscopy was vital to localize the lesion.

Early recognition and management are particularly warranted in patients with hemosuccus pancreaticus as the risk of mortality is up to 90% in untreated cases [18]. The treatment is largely based on the location and size of the involved peripancreatic artery. If the artery is localized and the size is small, stent or coil angiographic embolization is the initial therapeutic choice to achieve hemostasis, with a procedural success rate ranging from 79% to 100% [19]. Recently, balloon-assisted coil embolization has shown remarkable outcomes in this regard. However, it is notable that the embolotherapy has been associated with complications such as bowel ischemia, splenic infarct, infected aneurysm, and organ ischemia.

Surgical intervention is indicated for hemorrhage from relatively large-caliber arteries, especially in cases with failed embolization, uncontrolled bleeding, and persistent hemodynamic compromise [20]. Pancreatic duct ligature, pseudoaneurysm exclusion with arterial-end ligation, resection of the pancreas, and pancreatic pseudocyst drainage are common surgical options. Although aneurysmal exclusion is safe in most cases, it is not recommended in patients with pseudoaneurysms arising from the gastroduodenal and pancreaticoduodenal arteries due to a high recurrence rate of bleeding [20]. In the present case, coil embolization was initially unsuccessful to limit the enlarging vascular mass. The patient was deemed high risk for surgery due to the extraordinary projected mortality. Eventually, he and his family refused the embolization procedure, which led to the unfortunate outcome.

## Conclusions

Hemosuccus pancreaticus is an unusual, obscure cause of upper gastrointestinal bleeding. The diagnosis of this disease is often difficult to establish; however, several cross-sectional imaging and endoscopic modalities are used to detect the lesion with variable sensitivity and specificity. CTA has a higher diagnostic yield and can be used to rapidly confirm the diagnosis in most cases. Side-viewing duodenoscopy, as in the present case, should also be considered in these patients. Early interventional radiology and surgical procedures carry paramount importance in reducing the mortality rate. Future research should aim to frame standard guidelines for diagnosis and management in these patients.

## References

[1] Lower WE, Farrell JT (1931). Aneurysm of splenic artery: report of case and review of the literature. Arch Surg.

[2] Sandblom P (1970). Gastrointestinal hemorrhage through the pancreatic duct. Ann Surg.

[3] Maheshwaran MU, Sathyanesan J, Ramasamy S, Palaniappan R, Manoharan G (2016). Hemosuccus pancreaticus: 18-year experience from a tertiary care GI bleed centre in India. HPB.

[4] Kapoor S, Rao P, Pal S, Chattopadhyay TK (2004). Hemosuccus pancreaticus: an uncommon cause of gastrointestinal hemorrhage. A case report. JOP.

[5] Suter M, Doenz F, Chapuis G, Gillet M, Sandblom P (1995). Haemorrhage into the pancreatic duct (Hemosuccus pancreaticus): recognition and management. Eur J Surg.

[6] Dinu F, Deviere J, Van Gossum A, Golzarian J, Dussaussois L, Delhaye M, Cremer M (1998). The wirsungorrhagies: causes and management in 14 patients. Endoscopy.

[7] Han B, Song ZF, Sun B (2012). Hemosuccus pancreaticus: a rare cause of gastrointestinal bleeding. Hepatobiliary Pancreat Dis Int.

[8] El Hamel A, Parc R, Adda G, Bouteloup PY, Huguet C, Malafosse M (1991). Bleeding pseudocysts and pseudoaneurysms in chronic pancreatitis. Br J Surg.

[9] Risti B, Marincek B, Jost R, Decurtins M, Ammann R (1995). Hemosuccus pancreaticus as a source of obscure upper gastrointestinal bleeding: three cases and literature review. Am J Gastroenterol.

[10] Kuruma S, Kamisawa T, Tu Y, Egawa N, Tsuruta K, Tonooka A, Funata N (2009). Hemosuccus pancreaticus due to intraductal papillary-mucinous carcinoma of the pancreas. Clin J Gastroenterol.

[11] Inayat F, Ullah W, Hussain Q, Hurairah A (2017). Dieulafoy's lesion of the oesophagus: a case series and literature review. BMJ Case Rep.

[12] Yu P, Gong J (2018). Hemosuccus pancreaticus: a mini-review. Ann Med Surg (Lond).

[13] Inayat F, Amjad W, Hussain Q, Hurairah A (2018). Dieulafoy's lesion of the duodenum: a comparative review of 37 cases. BMJ Case Rep.

[14] Levy MJ, Wong Kee Song LM, Farnell MB, Misra S, Sarr MG, Gostout CJ (2008). Endoscopic ultrasound (EUS)-guided angiotherapy of refractory gastrointestinal bleeding. Am J Gastroenterol.

[15] Adler DG, Petersen BT, Gostout CJ (2004). Hemosuccus pancreaticus. Gastrointest Endosc.

[16] Koizumi J, Inoue S, Yonekawa H, Kunieda T (2002). Hemosuccus pancreaticus: diagnosis with CT and MRI and treatment with transcatheter embolization. Abdom Imaging.

[17] Singh-Bhinder N, Kim DH, Holly BP (2017). ACR Appropriateness Criteria® nonvariceal upper gastrointestinal bleeding. J Am Coll Radiol.

[18] Sreekantamurthy GG, Khan MJ, Kumar TSR (2017). Hemosuccus pancreaticus in chronic pancreatitis-a rare cause of upper GI bleed: a case report and review of literature. JOP.

[19] Rammohan A, Palaniappan R, Ramaswami S (2013). Hemosuccus pancreaticus: 15-year experience from a tertiary care GI bleed centre. ISRN Radiol.

[20] Sethi H, Peddu P, Prachalias A, Kane P, Karani J, Rela M, Heaton N (2010). Selective embolization for bleeding visceral artery pseudoaneurysms in patients with pancreatitis. Hepatobiliary Pancreat Dis Int.

